# Lateral Collateral Ligament Reconstruction Using the Triceps Brachii Fascia for Posterolateral Rotatory Instability in Cubitus Varus: A Report of Two Cases

**DOI:** 10.7759/cureus.54530

**Published:** 2024-02-20

**Authors:** Tatsuya Ito, Yutaka Mifune, Atsuyuki Inui, Hanako Nishimoto, Ryosuke Kuroda

**Affiliations:** 1 Department of Orthopaedic Surgery, Kobe University Graduate School of Medicine, Kobe, JPN

**Keywords:** reconstruction, triceps fascia, cubitus varus, posterolateral rotatory instability, elbow instability

## Abstract

A combination of osteotomy and ligament reconstruction is recommended for posterolateral rotatory instability (PLRI) with large cubitus varus deformities. There is a lack of reports regarding ligament donor selection for ligament reconstruction of PLRI with cubitus varus.

Two cases of PLRI with cubitus varus have been described. In case one, a 40-year-old woman presented with left elbow pain. She had a cubitus varus deformity, resulting from a childhood elbow fracture. Radiographs showed an 18-degree cubitus varus deformity. A lateral closing wedge osteotomy and double plate osteosynthesis were performed. The lateral collateral ligament (LCL) was reconstructed with autologous triceps fascia. Postoperative radiographs confirmed correction with 10 degrees of the carrying angle (CA). Bone union at the osteotomy site occurred six months later with excellent results. In case two, a 45-year-old man presented an arm with persistent right elbow instability with cubitus varus deformity. This was due to a childhood supracondylar fracture of the right humerus. Radiographs showed a cubitus varus deformity of 25 degrees on the right. The surgical procedure included a lateral wedge osteotomy, double plate fixation, and LCL reconstruction with autologous triceps fascia. Postoperative radiographs confirmed a corrected CA of 5 degrees. Bone union was achieved at the six-month follow-up with satisfactory results.

The use of triceps fascia for LCL reconstruction for PLRI due to cubitus varus would provide a minimally invasive and reasonable treatment option.

## Introduction

A cubitus varus deformity of the elbow joint can cause delayed-onset posterolateral rotatory instability (PLRI). The instability typically develops two to three decades after the onset of the osseous deformity. The pathophysiology of PLRI due to cubitus varus is explained in the literature [1]. A malunion of childhood elbow fractures, such as supracondylar fractures of the humerus, results in chronic varus stress on the lateral collateral ligament complex of the elbow. This stress is induced by the medial displacement of the mechanical axis (wrist to shoulder) and the displacement of the triceps force vector, creating a repetitive external rotatory torque on the ulna. Over time, this chronic varus stress leads to laxity or rupture of the lateral collateral ligament complex of the elbow. It ultimately results in instability 20 to 30 years later. There are not enough studies on the treatment of PLRI caused by cubitus varus in adults.

Most cases of cubitus varus remain asymptomatic for an extended period. Therefore, there is a lack of consensus regarding the timing and necessity of preventive surgery [1]. In children, the most common reason for surgery for cubitus varus is cosmetic deformity [2]. In adults, surgery has been considered when symptomatic conditions such as PLRI or ulnar nerve symptoms become apparent [1,3-5] or when there is a cosmetic problem [6].

Several surgical options are available to treat PLRI caused by cubitus varus, including standalone osteotomy, standalone ligament reconstruction, or a combination of both procedures. There are several options for potential donors to undergo ligament reconstruction surgery. These options include the palmaris longus, triceps, gracilis, and semitendinosus tendons [1,3-5]. The purpose of this report is to describe the outcomes of two cases of PLRI induced by cubitus varus treated with osteotomy and ligament reconstruction using the triceps fascia.

## Case presentation

Case one

**Figure 1:**
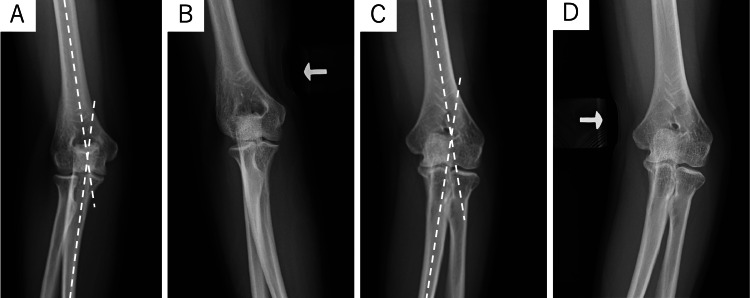
Preoperative radiographs of case one (A) Right AP radiograph. The CA was 12 degrees in the right upper extremity. (B) The right elbow showed no instability during varus stress testing. (C) Left AP radiographs showed an 18-degree cubitus varus deformity. (D) Left AP radiograph under varus stress. The left elbow showed 8 degrees of varus instability.

**Figure 2:**
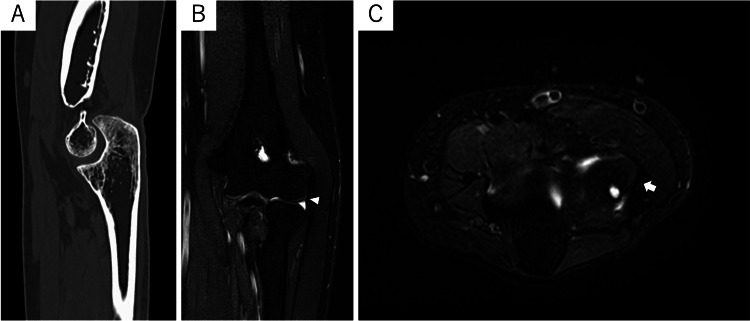
Preoperative CT and MRI scans of the left elbow of case one (A) No osteoarthritic changes were found in the CT scans. (B) The coronal T2-weighted fat suppression MRI showed a normal signal intensity of the LCL (white arrowhead). (C) The axial T2-weighted fat suppression MRI showed a normal signal intensity of the proximal attachment of the lateral collateral ligament (LCL) (white arrow).

A 40-year-old woman presented to our institution's outpatient clinic with pain in the left elbow during movements. She had suffered a left elbow fracture in childhood and received conservative treatment. A physical examination revealed an obvious cubitus varus deformity of the left elbow joint, with a limited range of motion (-20 degrees extension and 145 degrees flexion). During the stress test, pain and instability were observed on the lateral side of the left elbow. The PLRI test [7] for the left elbow was positive. X-rays showed an 18-degree cubitus varus deformity, compared to the carrying angle (CA) of 12 degrees in the right upper extremity. The right elbow showed no instability during varus stress testing, while the left elbow showed 8-degree varus instability (Figure 1). The preoperative Mayo Elbow Performance Score (MEPS) was 65. The CT scans showed no signs of osteoarthritic changes. The MRI showed the normal signal intensity of the lateral collateral ligament (LCL) on the T2-weighted fat suppression image (Figure 2). Based on these findings, the diagnosis was PLRI caused by cubitus varus. The patient was scheduled for surgical treatment, which included corrective humeral osteotomy and ligament reconstruction surgery. 

**Figure 3:**
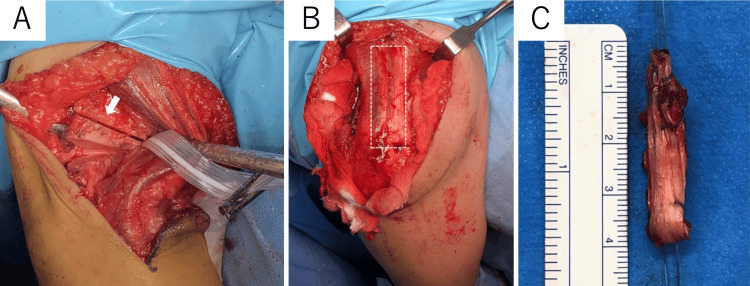
Intraoperative photographs of case one (A) A lateral closing wedge osteotomy was performed, and the osteotomy site is indicated by the white arrow. (B) The dashed line indicates where the triceps fascia was harvested. (C) The triceps fascia is folded.

**Figure 4:**
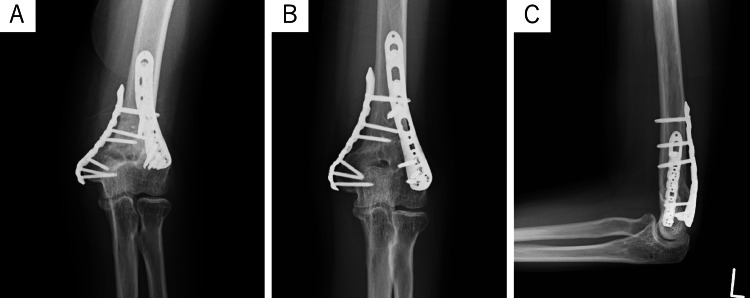
Postoperative radiographs of case one (A) The postoperative radiographs indicated that the left elbow had been corrected with a 10-degree carrying angle (CA). (B, C) The osteotomy site had achieved bone union after six months.

To expose the left elbow, a posterior approach was utilized, and a 20-degree lateral closing wedge osteotomy was performed. The angle was determined based on the healthy side's CA (Figure 3A). To stabilize the osteotomy site, double plates were used on the distal humerus. A 3x10 cm triceps fascia was harvested, folded in two, and utilized to reconstruct the LCL with interference screws (Figures 3B, 3C). The fascia was harvested from the middle one-third of the triceps muscle, preserving the enthesis area. After harvesting, the remaining fascia on both sides was sutured as much as possible. Postoperative X-rays revealed that the left elbow was corrected, with a CA of 10 degrees (Figure 4A). After the surgery, a cast was applied for three weeks. From the fourth week, active and active-assisted range of motion exercises of the elbow were initiated, while varus stress was strictly prohibited. Starting from the eighth week, a full range of motion was permitted, and the initiation of strengthening exercises was gradually allowed. The osteotomy site achieved bone union after six months (Figures 4B, 4C). At the one-year follow-up, the left elbow joint exhibited a range of motion with zero degrees of extension and 140 degrees of flexion, with 10 degrees of the CA. No postoperative complications were reported. One year after the operation, the MEPS was 100, indicating a significant improvement.

Case two

**Figure 5:**
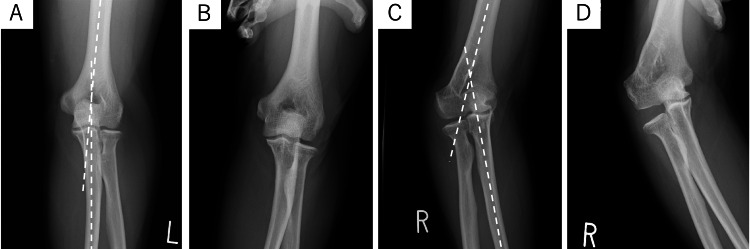
Preoperative radiographs of case two (A) Left AP radiograph. The carrying angle (CA) was 5 degrees in the left upper extremity. (B) The left elbow showed no instability on varus stress testing. (C) Right AP radiographs showed a cubitus varus deformity of 25 degrees. (D) Right AP radiograph under varus stress. The right elbow showed 15 degrees of varus instability.

**Figure 6:**
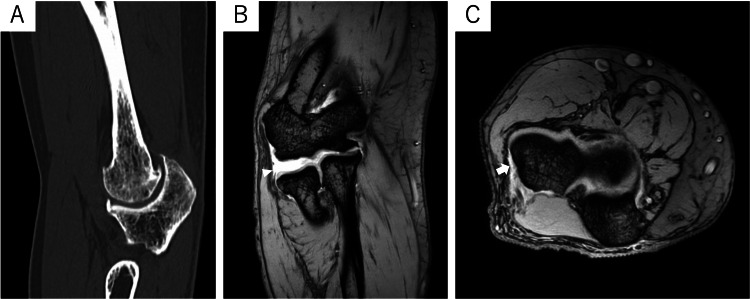
Preoperative CT and MRI scans of the right elbow of case two (A) CT scans showed osteoarthritic changes in the right ulnohumeral joint. (B) The coronal T2*-weighted MRI scan showed high-intensity signal enhancement in the lateral collateral ligament (LCL), indicative of a rupture (white arrowhead). (C) Axial T2*-weighted MRI showed high-intensity signal enhancement of the proximal attachment of the LCL (white arrow).

A 45-year-old male presented to our outpatient clinic with a persistent sense of instability in his right elbow over several years. He had a history of a right humeral supracondylar fracture and received conservative treatment. A physical examination revealed a cubitus varus deformity of the right elbow joint and limited range of motion (-5 degrees extension and 135 degrees flexion). During the varus stress test, pain and instability were observed on the lateral side of the right elbow. The PLRI test resulted positive. Plain X-rays revealed a cubitus varus deformity of 25 degrees on the right, compared to the CA of 5 degrees on the unaffected left upper extremity. The varus stress test did not show any changes in CA on the left, while on the right, there was a 15-degree varus instability (Figure 5). The preoperative MEPS was 60. CT scans revealed osteoarthritic changes in the right ulnohumeral joint, and an MRI showed a high-intensity signal change in the LCL in T2*-weighted images (Figure 6). The patient was diagnosed with PLRI due to cubitus varus, which required corrective humeral osteotomy and ligament reconstruction surgery.

**Figure 7:**
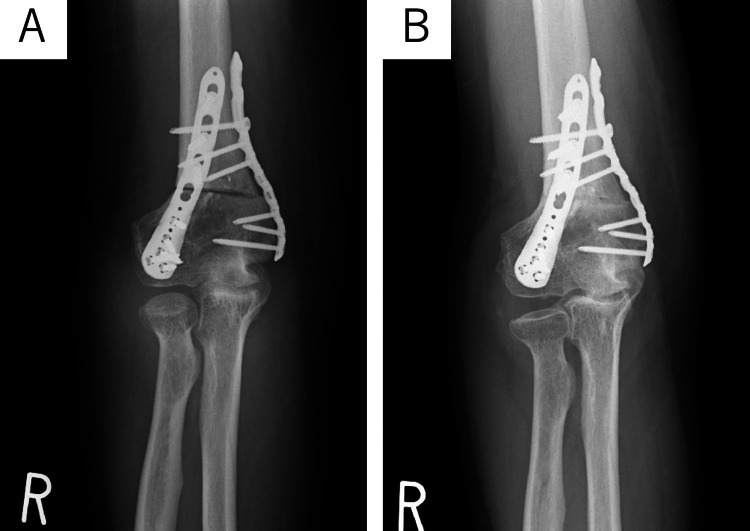
Postoperative radiograph of case two (A) Postoperative radiographs confirmed a corrected carrying angle (CA) of 5 degrees. (B) The osteotomy site had achieved bone union and CA was preserved at six months.

A posterior approach was used to perform a 30-degree lateral closing wedge osteotomy and double plate fixation. The triceps fascia was divided and used for LCL reconstruction with interference screws. Postoperative radiographs confirmed a corrected CA of 5 degrees (Figure 7A). A cast was applied for three weeks postoperatively and the rehabilitation protocol was the same as in case one. At the six-month follow-up, bone union was achieved and the range of motion of the right elbow joint was zero degrees of extension and 125 degrees of flexion. Plain radiographs showed a preserved CA of 5 degrees in the right elbow (Figure 7B). There were no postoperative complications. The MEPS at two years postoperatively improved from 60 preoperatively to 85.

## Discussion

This case report describes two cases of PLRI for cubitus varus deformity and demonstrates favorable short-term results with a combination of osteotomy and ligament reconstruction using the triceps fascia. Most cases of cubitus varus remain asymptomatic for long periods of time, so there are few reports of PLRI due to cubitus varus. There is no consensus on the timing and necessity of prophylactic surgery for cubitus varus [1]. For those with mild pain or no symptoms, treatment with medication or the use of a brace should be considered. Surgery is generally reserved for symptomatic cases, such as PLRI or ulnar nerve symptoms [1,3-5]. It is known that chronic PLRI often does not respond to conservative measures [8]. Therefore, our institution performs surgery for PLRI due to cubitus varus at the patient's request.

Abe et al. reported a 16-year-old male patient with PLRI due to cubitus varus with a history of supracondylar fracture of the distal humerus [3]. The patient underwent ligament repair and lateral closing wedge osteotomy. After 10 months, there was no instability, and the preoperative motion remained the same. 

O'Driscoll et al. conducted a multicenter study of 24 patients with 25 elbows presenting with posterolateral instability [1]. Of these 25, 22 had cubitus varus following a pediatric elbow fracture and three had congenital cubitus varus. Instability manifested two to three decades after the initial deformity. The average angle of varus deformity was 15 degrees. Of the 22 cases that underwent surgery, seven underwent ligament reconstruction and osteotomy, 10 underwent ligament reconstruction alone, four underwent osteotomy alone, and one underwent arthroplasty. Details of the grafts used were not reported. At a mean of three years after surgery, 19 of the 22 patients had good or excellent results. 

Patino et al. reported two patients with PLRI due to cubitus varus with a history of childhood elbow fractures [4]. A 29-year-old female patient underwent lateral wedge osteotomy with ligament reconstruction and the other patient, a 19-year-old female, underwent ligament reconstruction alone. In both cases, autologous palmaris longus tendons were used. The MEPS improved from 60 to 80 in four years in one patient and from 65 to 100 in two years in the other patient. 

Sachinis et al. reported a 54-year-old female patient with lateral ligament insufficiency due to cubitus varus resulting from a supracondylar fracture in childhood. The MRI showed a partial rupture of the common extensor tendon near its origin, and the LCL was intact. The patient underwent an open lateral ulnar collateral ligament augmentation technique using polyethylene tape and two bone anchors without osteotomy. The common extensor origin was also augmented and refixed to the lateral epicondyle. The MEPS improved from 50 to 100 over two years. The authors emphasized that this procedure does not correct humeroulnar alignment and was used alone because the patient required early active motion on her elbow. They also mentioned that it remains unclear whether the described procedure is equivalent to combined ligament reconstruction and osteotomy [5]. Clinical outcomes of past reports and present cases are summarized in Table 1.

**Table 1:** Clinical outcomes of past reports and present cases MEPS: Mayo Elbow Performance Score

Included studies	Surgical cases	Mean age (years)	Preoperative mean cubitus varus (degrees)	Cause of cubitus varus	Surgical technique	Type of graft	Preoperative MEPS	Postoperative MEPS
Abe et al. [3]	1	16	Not reported	Pediatric elbow fracture (1)	Ligament repair and osteotomy (1)	No use	Not reported	Not reported
O'Driscoll et al. [1]	22	34	15 (0-35)	Pediatric elbow fracture (19), congenital deformity (3)	Ligament reconstruction and osteotomy (7), ligament reconstruction alone (10), osteotomy alone (4), arthroplasty (1)	Not reported (22)	59 (30-85)	87 (50-100)
Patiño et al. [4]	2	24	15 (10-20)	Pediatric elbow fracture (2)	Ligament reconstruction and osteotomy (1), ligament reconstruction alone (1)	Palmaris longus (2)	62.5 (60-65)	90 (80-100)
Sachinis et al. [5]	1	54	25	Pediatric elbow fracture (1)	Ligament augmentation (1)	Polyethylene tape (1)	Not reported	Not reported
Present cases	2	42.5	21.5 (18-25)	Pediatric elbow fracture (2)	Ligament reconstruction and osteotomy (2)	Triceps fascia (2)	62.5 (60-65)	92.5 (85-100)
Overall	28	34	15.9	Pediatric elbow fracture (25/28), congenital deformity (3/28)	Ligament repair and osteotomy (1/28), ligament reconstruction and osteotomy (10/28), ligament reconstruction alone (11/28), osteotomy alone (4/28), arthroplasty (1/28), ligament augmentation (1/28)	Not reported (22/27), palmaris longus (2/27), polyethylene tape (1/27), triceps fascia (2/27)	59.5 (n=26)	87.7 (n=26)

The surgical methods for PLRI can be listed as follows: ligament reconstruction or repair alone, osteotomy alone, osteotomy combined with ligament repair, and osteotomy combined with ligament reconstruction. O'Driscoll et al. recommend a combination of osteotomy and ligament reconstruction for large cubitus varus deformities (greater than 15 degrees) due to the high risk of failure associated with either procedure alone [1]. There is a report indicating that performing osteotomy alone for cosmetic concerns in cubitus varus has led to the occurrence of new PLRI [9]. This may suggest the potential needed to combine osteotomy with ligament reconstruction surgery. In both cases presented in this report, we performed osteotomy combined with ligament reconstruction due to a varus deformity that exceeded 15 degrees.

Various osteotomy procedures have been reported to correct cubitus varus. These procedures can be divided into four groups: lateral closing wedge osteotomy, dome osteotomy, complex osteotomy, and distraction osteogenesis [10]. However, there are relatively few reports on osteotomy for adult cubitus varus compared to children [1,3-5,9-14]. Cubitus varus typically involves an internal rotation deformity, but it is unclear whether correcting this deformity is necessary for a successful outcome [15]. Furthermore, there is a risk of non-union in adults due to a reduced bone contact area. Our lateral closing wedge osteotomy, which does not correct internal rotation deformity, with double plate fixation has achieved rigid fixation and early range of motion exercise, resulting in favorable surgical outcomes and bone union.

Most LCL reconstructions use palmaris longus tendon grafts, followed by triceps, synthetic, gracilis tendon, and semitendinosus tendon grafts [16,17]. Badhrinarayanan et al. [16] found no significant difference in the rate of recurrent instability between different graft types. Biomechanical data supports the equivalence of the palmaris longus tendon and triceps fascia in terms of ultimate failure strength and stiffness. The study by Martin et al. showed that harvesting from the triceps fascia does not decrease triceps strength [18]. When using triceps grafts, graft harvesting, ligament reconstruction, and rigid osteosynthesis with plates, it can be performed through the same incision. Additionally, this approach offers flexibility in graft size [18]. We believe that using triceps fascia is less invasive and more practical than other autograft methods.

## Conclusions

For PLRI with cubitus varus, ligament reconstruction using the triceps fascia allows osteotomy, graft harvest, ligament reconstruction, and double plate fixation through a single skin incision. Our procedure resulted in favorable surgical outcomes and bone union. The use of the triceps fascia for LCL reconstruction for PLRI due to cubitus varus would be a minimally invasive and reasonable treatment option.
